# Biosynthesis of medicinally important plant metabolites by unusual type III polyketide synthases

**DOI:** 10.1007/s11418-020-01414-9

**Published:** 2020-06-05

**Authors:** Ikuro Abe

**Affiliations:** Graduate School of Pharmaceutical Sciences, The University of Tokyo, 7-3-1 Hongo, Bunkyo-ku, Tokyo, 113-0033 Japan

**Keywords:** Biosynthesis, Enzyme, Type III polyketide synthase, Curcumin, Quinolone, Tropane alkaloid

## Abstract

Recent research progress on the “second generation” type III polyketide synthases is summarized. This class of enzymes catalyzes unusual condensation chemistries of CoA thioesters to generate various core structures of medicinally important plant secondary metabolites, including the R_1_–C–R_2_ scaffold of alkyl quinolones, curcuminoids, as well as the 8-azabicyclo[3.2.1]octane ring of tropane alkaloids. The discovery of this fascinating enzyme superfamily provides excellent opportunities for the manipulation of the enzyme reactions to expand the supply of natural and unnatural molecules for future drug development.

**Electronic supplementary material:**

The online version of this article (10.1007/s11418-020-01414-9) contains supplementary material, which is available to authorized users.

## Introduction

**Fig. 1:**
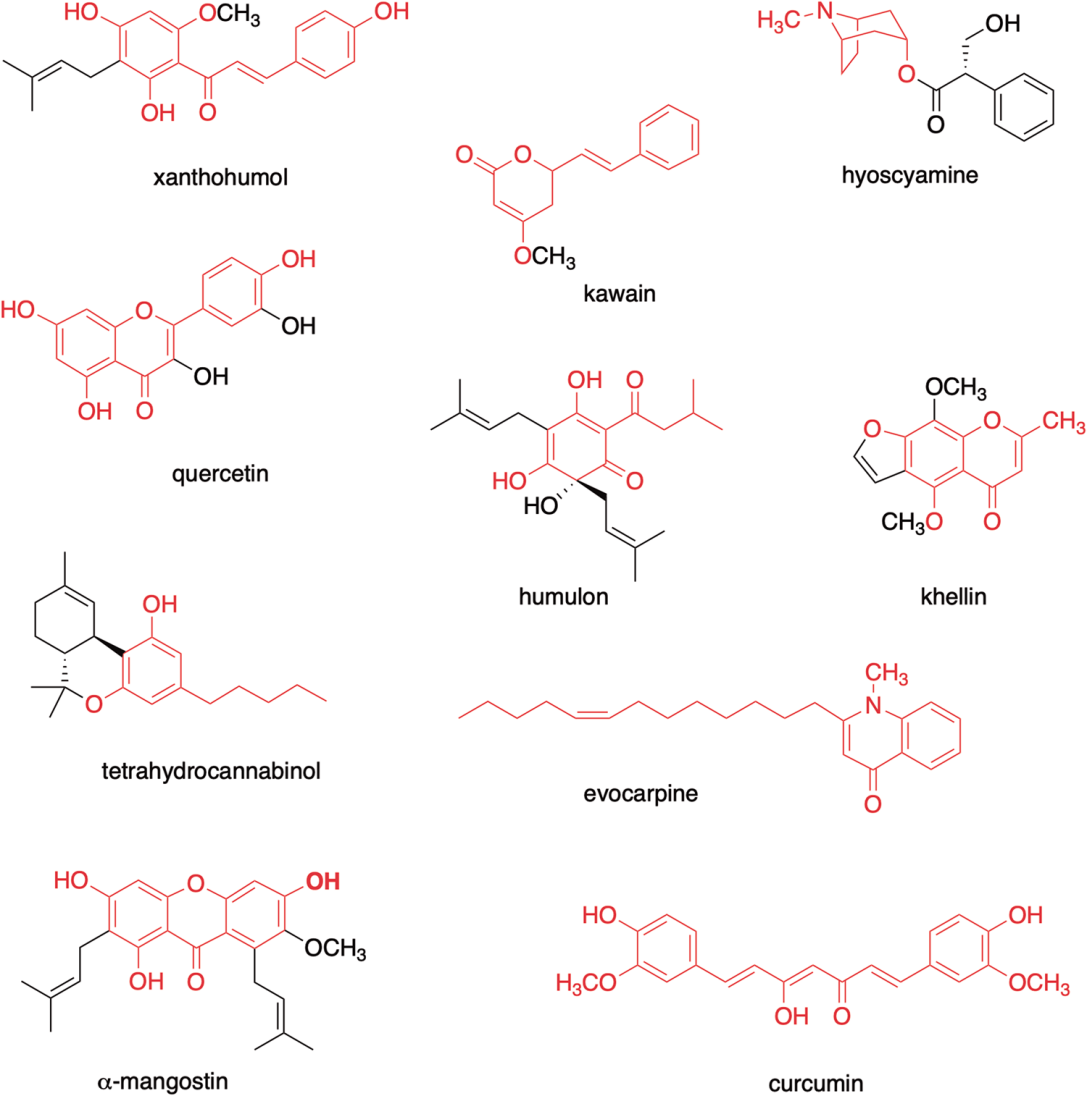
Typical plant secondary metabolites produced by plant type III PKSs

**Fig. 2:**
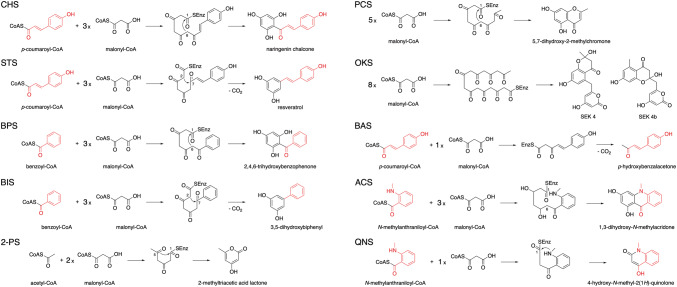
Typical reactions catalyzed by plant type III PKSs

The type III polyketide synthase (PKS) superfamily enzymes generate incredibly diverse core structures of medicinally important plant secondary metabolites, including flavonoids, stilbenes, chromones, pyrones, phloroglucinols, resorcinols, xanthones, acridones, and quinolones (Figs. 1, 2) [1–5]. For example, chalcone synthase **(**CHS) and stilbene synthase (STS) are plant-specific typical type III PKSs that accept *p*-coumaroyl-CoA as the starter substrate to catalyze three successive condensations with malonyl-CoA to generate naringenin chalcone and resveratrol, respectively. These enzymes utilize different (Claisen-type or decarboxylative aldol-type) cyclizations of an enzyme-bound, common linear poly-β-keto intermediate (Fig. 2). Recent biochemical and structural investigations have revealed that the homodimeric superfamily enzymes possess a highly homologous overall structure, with a conserved Cys-His-Asn catalytic triad as well as a characteristic CoA-binding tunnel [1–5]. The enzyme reactions are initiated by loading of the starter substrate, polyketide chain elongation through decarboxylative condensation with the extender substrate, and termination by cyclization of the resulting intermediate, within a single active site. The prominent diversity of the functions of these highly homologous enzymes is attributed to the slight differences in the volume and architecture of the active site cavity, which determine the preference for the starter and extender substrates, the number of chain elongation reactions, and the mode of cyclization reactions [1–5].

This short review focuses on the recently reported unusual “second generation” type III PKS enzymes [5], which mediate the fascinating chemistry of condensation reactions of CoA thioesters to generate various core structures of medicinally important plant secondary metabolites. For example, in the biogenesis of evocarpine in Rutaceae plants, two functionally distinct type III PKSs cooperatively mediate the condensation of three substrates (R_1_CO–CoA, R_2_CO–CoA, and malonyl-CoA) to generate the R_1_–C–R_2_ core scaffold of the 2-alkylquinolone alkaloids [6]. This is also the case for the biosynthesis of the diarylheptanoid scaffold of curcumin in Zingiberaceae plants [7–9]. In even more interesting cases, a single enzyme catalyzes the condensations of three substrates, to achieve the one-pot biogenesis of the alkylquinolone [10] or curcuminoid [11, 12] scaffold. Finally, a recently discovered unusual type III PKS is responsible for the biogenesis of tropane alkaloids in Solanaceae plants, and produces tropinone with the 8-azabicyclo[3.2.1]octane scaffold from two molecules of malonyl-CoA and *N*-methyl-Δ^1^-pyrrolinium cation [13, 14]. These unusual enzymes have significantly expanded the catalytic repertoires of the type III PKS superfamily enzymes, and provide an excellent platform for manipulation of the enzyme reactions for the further production of medicinally important natural and unnatural molecules.

## Evocarpine and alkylquinolone alkaloids

**Fig. 3:**
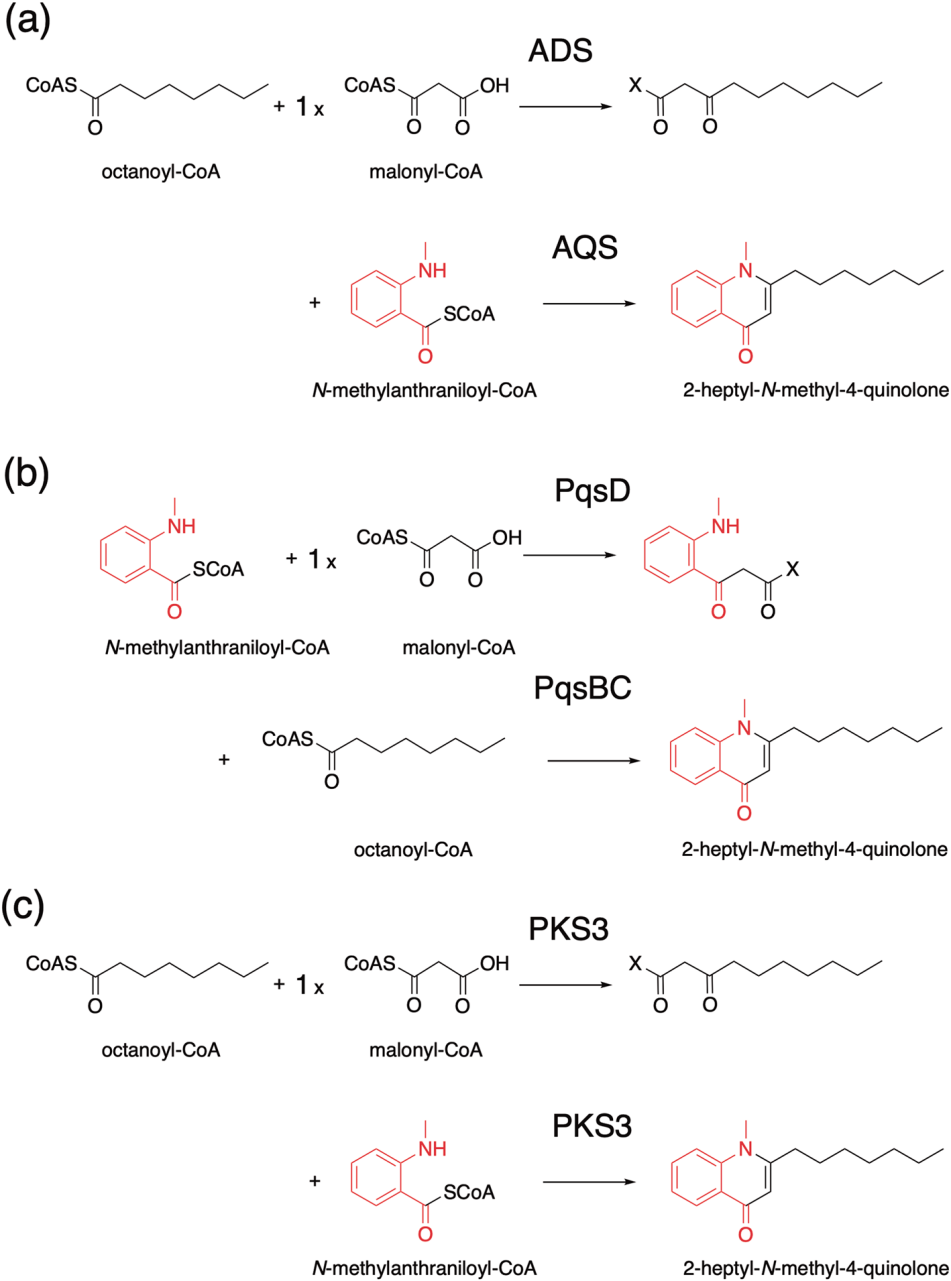
Proposed mechanism for the formation of 2-alkylquinolones: **a** by *E. rutaecarpa* ADS and AQS, **b** by *P. aeruginosa* PqsD and PqsBC, and **c** by *H. serrata* PKS3

2-Alkylquinolone (2AQ) alkaloids, such as evocarpine (Fig. 1), one of the active principle components of the traditional Chinese medicine Evodia fruit, are important medicinal natural products that exhibit prominent biological activities, including antibacterial, cytotoxic, and acetylcholinesterase-inhibiting activities [15, 16]. In the biosynthesis of evocarpine, the 2AQ scaffold is produced by the co-action of two novel type III PKSs, alkylquinolone synthase (AQS) and alkyldiketide-CoA synthase (ADS), in *Evodia rutaecarpa* (Rutaceae) [6]. AQS and ADS share 46–76% amino acid sequence identity with other plant type III PKSs and 61% identity with each other. In this case, these two enzymes mediate the condensation of three substrates: fatty acyl-CoA (C_8_–C_12_), *N*-methylanthraniloyl-CoA, and malonyl-CoA, to yield 2-alkyl-*N*-methyl-4-quinolones (Fig. 3a). Thus, ADS first generates an alkyldiketide-CoA by the decarboxylative condensation of fatty acyl-CoA and malonyl-CoA. After non-enzymatic hydrolysis, AQS then catalyzes the coupling of the diketide acid and *N*-methylanthraniloyl-CoA through C–C and C–N bond formations, thereby completing the synthesis of the 2AQ scaffold. This biosynthetic machinery is similar to that for curcumin production in Zingiberaceae plants [7–9], which will be discussed below.

Interestingly, bacteria also produce 2AQs as quorum-sensing signals [17]. However, the biosynthesis of the bacterial 2AQs (Fig. 3b) is totally distinct from that of the plant 2AQs. Thus, in *Pseudomonas aeruginosa*, the β-ketoacyl carrier protein synthase III (FabH)-like enzyme PqsD and the thioesterase PqsE first produce 2-aminobenzoyldiketide acid, by condensation of anthraniloyl-CoA with malonyl-CoA. Subsequently, the heterodimer of other FabH-like enzymes, PqsB and PqsC, catalyzes the decarboxylative condensation of a fatty acyl-CoA and the acid to construct the 2AQ scaffold [18–20]. The three FabH-like enzymes, PqsB, C, and D, exhibit less than 20% sequence identities with AQS and ADS. It is likely that the biosynthetic machineries of 2AQ in bacteria and plants evolved in a parallel, but independent fashion in *P. aeruginosa* and Rutaceae plants.

**Fig. 4:**
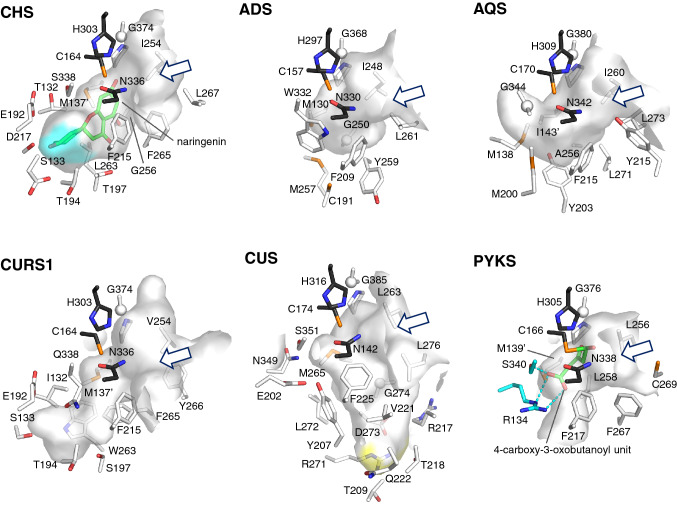
Active site cavities observed in the crystal structures of *Medicago sativa* CHS (PDB entry: 1CGK), *E. rutaecarpa* ADS (PDB entry: 5WX3), *E. rutaecarpa* AQS (PDB entry: 5WX4), *C. longa* CURS1 (PDB entry: 3OV2), *O. sativa* CUS (PDB entry: 3OIT), and *A. acutangulus* PKYS (PDB entry: 6J1M)

To investigate the structure–function relationship, the crystal structures of *E. rutaecarpa* AQS and ADS were solved at 2.2 and 1.8 Å resolutions, respectively (Fig. 4) [6]. The overall structures of AQS and ADS are nearly identical, and also highly homologous to those of other type III PKS superfamily enzymes, with the conserved Cys-His-Asn catalytic triad and most of the residues lining the active site cavity. In contrast, close examination of the AQS and ADS structures revealed the novel CoA-binding tunnel with Tyr215 in AQS and the unique and unprecedented active site geometry with Trp332 and Cys191 in ADS. These characteristic active site structures seem to be important for governing the specificities of the substrate/product and the unique catalytic functions of AQS and ADS, as further supported by site-directed mutagenesis. Notably, despite the lack of significant hydrophobicity in the CoA-binding tunnel, AQS accommodates alkyldiketide acid (C_6_–C_14_) as the extender to catalyze condensations with *N*-methylanthraniloyl-CoA. On the other hand, ADS can accommodate a fatty acyl-CoA (C_8_–C_12_) as the starter substrate to yield alkyldiketide-CoAs.

## Curcumin and curcuminoids

**Fig. 5:**
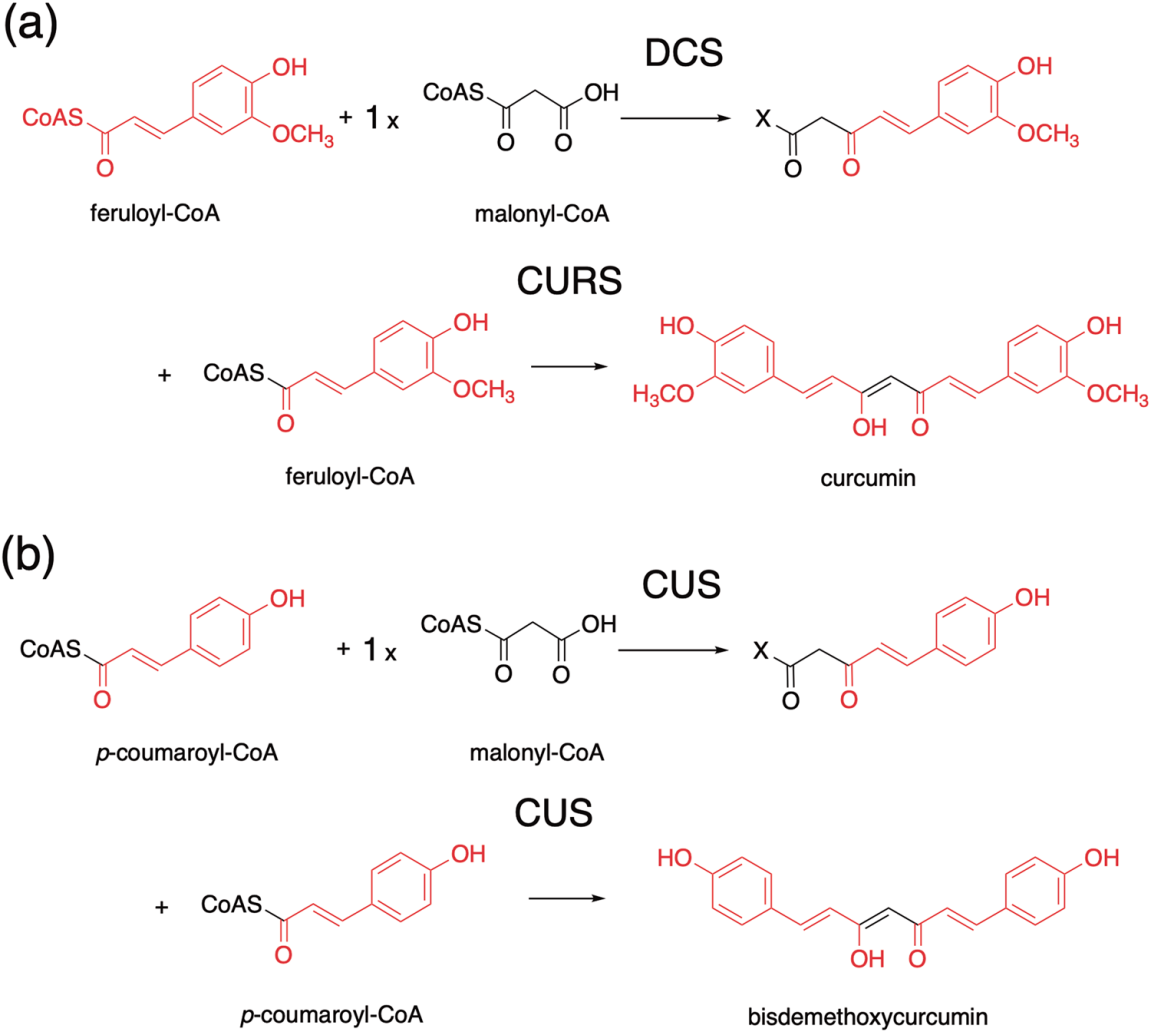
Proposed mechanism for the formation of **a** curcumin by *C. longa* DCS and CURS, and **b** bisdemethoxycurcumin by *O. sativa* CUS

In a similar fashion to the 2AQ biosynthesis, the diarylheptanoid scaffold of curcumin is produced by the co-action of two type III PKSs, curcumin synthase (CURS) and diketide-CoA synthase (DCS), in turmeric *Curcuma longa* (Zingiberaceae) (Fig. 5a) [7–9]. CURS and DCS share 47–63% sequence identity with other plant type III PKSs and 63% identity with each other. Thus, diketide-CoA synthase (DCS) first catalyzes the decarboxylative coupling of feruloyl-CoA with malonyl-CoA to generate feruloyldiketide-CoA. In contrast, CURS1 catalyzes the hydrolysis of the diketide-CoA and the subsequent condensation of the resulting diketide acid extender with another feruloyl-CoA, leading to the biogenesis of the C_6_–C_7_–C_6_ diarylheptanoid scaffold of curcumin. Interestingly, the turmeric plant expresses three CURS isozymes (CURS1-3) [8]. The X-ray crystal structure of *C. longa* CURS1 solved at 2.3 Å resolution revealed a characteristic active site structure with an unusual hydrophobic pocket in the CoA-binding tunnel, which is formed by the different orientation of the gatekeeper Phe265 and the characteristic replacement of the active site Ser338 with Gln338 (Fig. 4) [9].

On a particular note, the curcuminoid synthase (CUS) reported from the genome of the rice plant *Oryza sativa* (Poaceae) is an extremely interesting enzyme, in that even a single type III PKS can catalyze the one-pot construction of the curcuminoid scaffold from *p*-coumaroyl-CoA and malonyl-CoA (Fig. 5b) [11]. In this case, the bifunctional CUS first generates *p*-coumaroyldiketide-CoA from *p*-coumaroyl-CoA by condensation with malonyl-CoA, which is followed by decarboxylative coupling with another *p*-coumaroyl-CoA, thereby generating the diarylheptanoid scaffold of bisdemethoxycurcumin. One of the most interesting points here is how this structurally simple enzyme precisely controls the order of substrate loading at the active site (the bulky *p*-coumaroyl-CoA first, the small malonyl-CoA second, and finally the bulky *p*-coumaroyl-CoA again) during the successive enzyme reactions.

The X-ray crystal structure solved at 2.5 Å resolution revealed the unprecedented active site architecture of *O. sativa* CUS (Fig. 4) [12]. The huge downward-expanding active site has sufficient room to simultaneously accommodate the two bulky coumarate and malonate substrates. Another interesting feature is the presence of a H_2_O molecule, fixed by the hydrogen bonding Glu202-Tyr207-H_2_O-Asn142-Ser351 residues, at the catalytic center in the proximity of Cys174. CUS is likely to employ this putative nucleophilic H_2_O to interrupt the polyketide chain elongation, and thus yield *p*-coumaroyldiketide acid by subsequent thioester bond cleavage. This intermediate is then loaded into the downward pocket, and finally, the second condensation with another *p*-coumaroyl-CoA completes the one-pot synthesis of bisdemethoxycurcumin.

In addition to *O. sativa* CUS, the recently reported PKS3 from the Chinese club moss *Huperzia serrata* (Lycopodiaceae) [10] is another fascinating type III PKS that also catalyzes the one-pot condensation of feruloyl-CoA (or *p*-coumaroyl-CoA or cinnamoyl-CoA) and malonyl-CoA to yield a series of curcuminoids (Fig. 5b). Furthermore, the promiscuous *H. serrata* PKS3 also accepts *N*-methylanthraniloyl-CoA, fatty acyl-CoA, and other CoA thioesters as substrates to perform the one-pot synthesis of the R_1_–C–R_2_ scaffolds of natural and unnatural 2-alkylquinolones and 1,3-diketones (Fig. 3c). The catalytic versatility of the *O. sativa* CUS and *H. serrata* PKS3 enzymes thus provides excellent opportunities for the development of useful biocatalysts to supply structurally diverse novel curcuminoids, 2-alkylquinolones, and 1,3-diketones for future drug discovery.

## Tropinone and tropan alkaloids

**Fig. 6:**
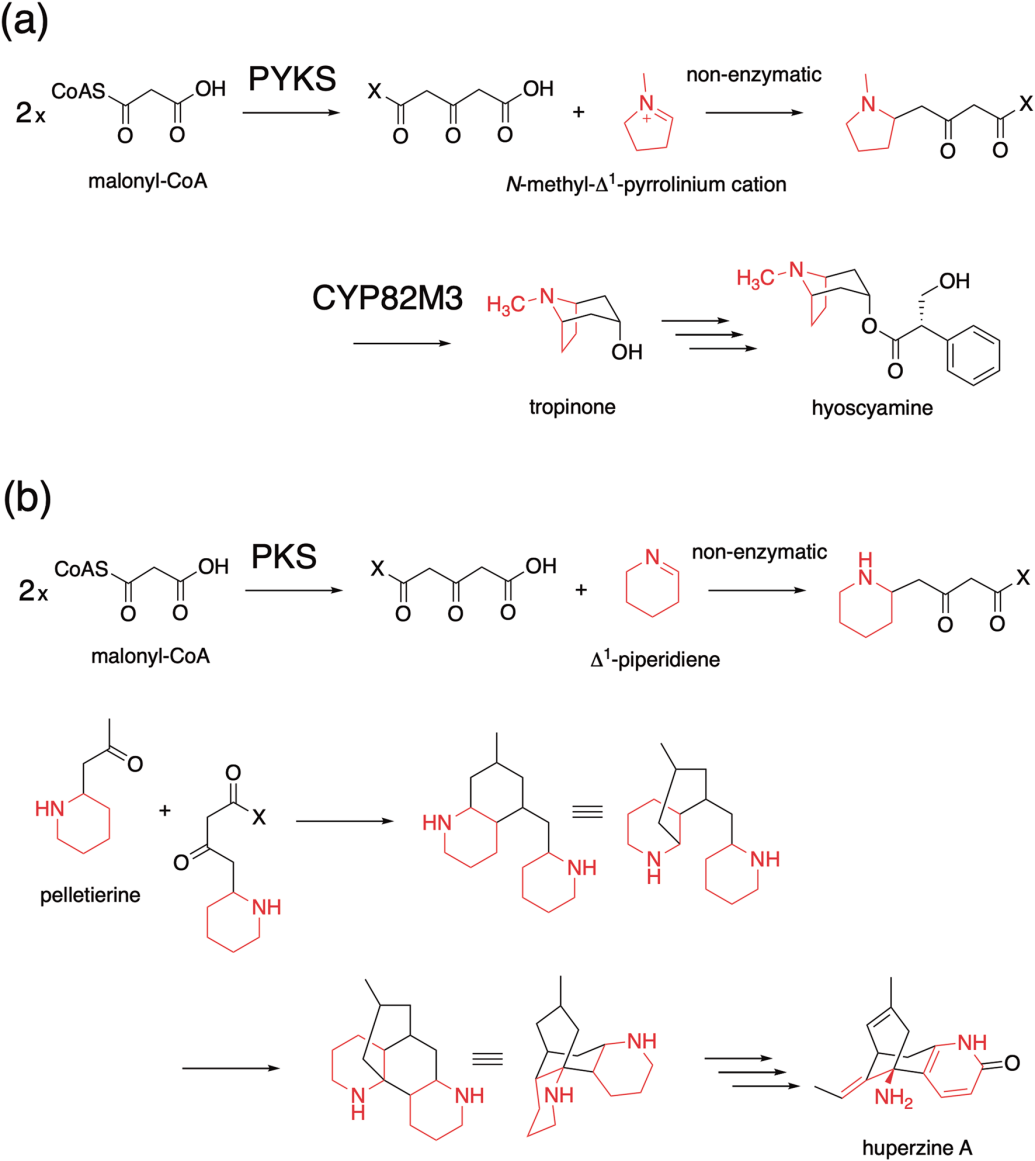
Proposed mechanism for the formation of **a** tropinone by *A. acutangulus* PYKS and CYP82M3, and **b** hypothetical biosynthetic pathway for lycopodium alkaloids

Tropane alkaloids produced by Solanaceae and Erythroxylaceae plants are medicinally important plant metabolites with a characteristic 8-azabicyclo[3.2.1]octane scaffold. It was recently discovered that tropinone, the precursor of hyoscyamine and scopolamine, is biosynthesized by the collaboration of another unusual type III PKS, pyrrolidine ketide synthase (PYKS), with a cytochrome P450 monooxygenase-mediated cyclization reaction, in *Atropa belladonna*, *Datura stramonium*, and *Anisodus acutangulus* (Fig. 6a) [13, 14]. In this case, PYKS was proposed to catalyze only one decarboxylative condensation of two molecules of malonyl-CoA to yield the diketide 4-carboxy-3-oxobutanoyl-CoA, which then undergoes hydrolysis and a “non-enzymatic” Mannich-like condensation with an *N*-methyl-Δ^1^-pyrrolinium cation to generate the racemic 4-(1-methyl-2-pyrrolidinyl)-3-oxobutanoic acid. Subsequently, the formation of the tropinone with the 8-azabicyclo[3.2.1]octane scaffold is catalyzed by P450 CYP82M3, through a stereo-specific oxidative cyclization reaction.

The X-ray crystal structures of *A. acutangulus* PYKS were solved at 2.0–2.5 resolution [14]. The overall structure of the homodimeric PYKS, with the conserved Cys166-His305-Asn338 catalytic triad and CoA-binding tunnel, is highly homologous to those of other type III PKSs; however, the active site cavity is significantly smaller (Fig. 4). The PYKS structures in complex with either 4-carboxy-3-oxobutanoyl-CoA in the CoA-binding tunnel or the 4-carboxy-3-oxobutanoyl diketide intermediate covalently bound to the catalytic Cys166 further suggested that PYKS is responsible for only the single condensation of two molecules of malonyl-CoA to produce the diketide 4-carboxy-3-oxobutanoyl-CoA. The proposed “non-enzymatic” Mannich-like condensation between the *N*-methyl-Δ^1^-pyrrolinium cation and 4-carboxy-3-oxobutanoic acid was also supported by experimental chemical reactions, in the presence or absence of PYKS. Notably, the biosynthesis is closely analogous to the legendary tropinone synthesis described by Sir Robert Robinson in 1917 [21]. This finding led to the recent development of a synthetic biology platform for the production of tropane alkaloids in a yeast system [22].

The discovery of PYKS for the tropane alkaloid biosynthesis in Solanaceae plants also suggests the possible involvement of similar type III PKSs in the biogenesis of lycopodium alkaloids in Lycopodiaceae plants [23]. This includes huperzine A, a potent acetylcholine esterase inhibitor and promising anti-Alzheimer disease drug candidate, from the Chinese club moss *H. serrata*. In this case, in a similar manner, a PYKS-like type III PKS first produces the diketide 4-carboxy-3-oxobutanoyl-CoA, which then undergoes the “non-enzymatic” coupling with Δ^1^-piperideine to yield 4-(2-piperidyl) acetoacetate (or its CoA or decarboxylated pelletierine) (Fig. 6b). Another AQS- and CURS-like type III PKS might be involved in the following decarboxylative coupling of 4-(2-piperidyl) acetoacetyl-CoA and pelletierine to produce phlegmarine, which is the biosynthetic precursor for lycopodium alkaloids in Lycopodiaceae plants.

## Conclusions

The discovery of the unusual “second generation” enzymes has significantly expanded the catalytic repertoire of the type III PKS superfamily enzymes. Further investigations of the structure–function relationships of these fascinating enzymes will provide excellent opportunities for manipulations of the enzyme reactions, for the further production of natural and unnatural medicinally important molecules for future drug development.

## Electronic supplementary material

Below is the link to the electronic supplementary material.

Supplementary file 1 (CDX 31 kb)

Supplementary file 2 (CDX 185 kb)

Supplementary file 3 (CDX 81 kb)

Supplementary file 4 (CDX 61 kb)

Supplementary file 5 (CDX 58 kb)
