# Correction to: Biosynthesis of medicinally important plant metabolites by unusual type III polyketide synthases

**DOI:** 10.1007/s11418-020-01424-7

**Published:** 2020-06-22

**Authors:** Ikuro Abe

**Affiliations:** Graduate School of Pharmaceutical Sciences, The University of Tokyo, 7-3-1 Hongo, Bunkyo-ku, Tokyo, 113-0033 Japan

## Correction to: Journal of Natural Medicines 10.1007/s11418-020-01414-9

The article Biosynthesis of medicinally important plant metabolites by unusual type III polyketide synthases, written by Ikuro Abe, was originally published electronically on the publisher’s internet portal on 05 June 2020 without open access. With the author(s)’ decision to opt for Open Choice the copyright of the article changed on 18 June 2020 to © The Author(s) 2020 and the article is forthwith distributed under a Creative Commons Attribution 4.0 International License (https://creativecommons.org/licenses/by/4.0/), which permits use, sharing, adaptation, distribution and reproduction in any medium or format, as long as you give appropriate credit to the original author(s) and the source, provide a link to the Creative Commons licence, and indicate if changes were made.

The original article was updated.

